# TYK2 promotes malignant peripheral nerve sheath tumor progression through inhibition of cell death

**DOI:** 10.1002/cam4.2386

**Published:** 2019-07-06

**Authors:** Wenjing Qin, Abigail Godec, Xiaochun Zhang, Cuige Zhu, Jieya Shao, Yu Tao, Xianzhang Bu, Angela C. Hirbe

**Affiliations:** ^1^ Division of Oncology, Department of Internal Medicine Washington University School of Medicine St. Louis Missouri; ^2^ School of Pharmaceutical Sciences Sun Yat‐Sen University Guangzhou China; ^3^ Siteman Cancer Center Washington University School of Medicine Saint Louis Missouri; ^4^ Cancer Center Biostatistics Shared Resource, Division of Public Health Sciences, Department of Surgery Washington University School of Medicine St. Louis Missouri

**Keywords:** cancer, MPNST, sarcoma, TYK2

## Abstract

**Background:**

Malignant peripheral nerve sheath tumors (MPNSTs) are aggressive sarcomas that arise most commonly in the setting of the Neurofibromatosis Type 1 (NF1) cancer predisposition syndrome. Despite aggressive multimodality therapy, outcomes are dismal and most patients die within 5 years of diagnosis. Prior genomic studies in our laboratory identified *tyrosine kinase 2* (*TYK2*) as a frequently mutated gene in MPNST. Herein, we explored the function of TYK2 in MPNST pathogenesis.

**Methods:**

Immunohistochemistry was utilized to examine expression of TYK2 in MPNSTs and other sarcomas. To establish a role for TYK2 in MPNST pathogenesis, murine and human TYK2 knockdown and knockout cells were established using shRNA and CRISPR/Cas9 systems, respectively.

**Results:**

We have demonstrated that TYK2 was highly expressed in the majority of human MPNSTs examined. Additionally, we demonstrated that knockdown of *Tyk2/TYK2* in murine and human MPNST cells significantly increased cell death in vitro. These effects were accompanied by a decrease in the levels of activated Stats and Bcl‐2 as well as an increase in the levels of Cleaved Caspase‐3. In addition, *Tyk2*‐KD cells demonstrated impaired growth in subcutaneous and metastasis models in vivo.

**Conclusion:**

Taken together, these data illustrate the importance of TYK2 in MPNST pathogenesis and suggest that the TYK2 pathway may be a potential therapeutic target for these deadly cancers.

## 1 INTRODUCTION

Malignant peripheral nerve sheath tumors are aggressive sarcomas that account for approximately 5% of all soft tissue sarcomas. Approximately, 50% of these tumors occur in patients with the Neurofibromatosis Type 1 (NF1) cancer predisposition syndrome, while the other half occur sporadically or as a secondary complication of radiation therapy.^1^, ^2^ In the setting of NF1, MPNSTs arise from malignant transformation of a benign precursor lesion, a plexiform neurofibroma.^3^ Initial treatment for MPNSTs typically involves surgery and radiation with or without chemotherapy.^4^ However, despite aggressive therapy, the recurrence rate is high and the vast majority of people with these cancers will die within 5 years of diagnosis.^5^, ^6^, ^7^, ^8^ Treatment for metastatic disease is limited to cytotoxic chemotherapy and clinical trials evaluating experimental therapies. As such, there is a pressing need to identify novel therapeutic targets.

Prior work from our laboratory identified *TYK2* as a gene mutated and frequently overexpressed in a subset of MPNSTs.^9^ Tyrosine kinase 2 (TYK2) is a member of the Janus kinase (JAK family),^10^ which includes three other members, JAK1‐3. These proteins are non‐receptor tyrosine kinases that are activated by auto or trans‐phosphorylation after a cytokine binds to its respective receptor.^11^, ^12^ Subsequently, JAKs activate signal transducers and activators of transcription proteins (STATs) which form homo‐/hetero dimers and then following phosphorylation, translocate into the nucleus to induce the transcription of target genes.^13^, ^14^ STAT protein family members have been shown to regulate oncogenic signaling in many tumor types often through the expression of downstream target genes such as cyclin D, Bcl‐2, and Bcl‐x, which regulate cell proliferation^15^, ^16^ and apoptosis^17^ of tumor cells. Herein, we have explored the role of TYK2 in MPNST pathogenesis. First, we have shown that TYK2 is expressed in over 60% of human MPNSTs. Second, knockdown of the gene in murine and human MPNST cells led to decreased phosphorylation of STAT proteins, decreased levels of Bcl2, and increased cell death in vitro. Finally, we showed that knockdown of the gene led to decreased tumor burden in a primary and metastatic xenograft model. Taken together, these data suggest that TYK2 is a potential therapeutic target for MPNSTs.

## 2 METHODS

### 2.1 Cell culture

Murine MPNST tumor lines expressing GFP‐Luciferase established previously in our laboratory from C57BL6/J *Nf1±;Trp53± (NPcis)*,^18^ and human MPNST 724 cell lines obtained from Jonathan Fletcher (Dana Farber Cancer Institute) were used for all in vitro and in vivo experiments. Cells were cultured in high‐glucose DMEM (Gibco Life Technologies, Grand Island, NY, USA), with 10% FBS (Gibco Life Technologies, Grand Island, NY, USA) and penicillin‐streptomycin (200 µg/mL).

### 2.2 Lentiviruses

Lentiviral *Tyk2* shRNA (TRCN0000236001, TRCN0000236003, TRC0000361657, MilliporeSigma, St. Louis, MO) or control *LacZ* shRNA (McDonnell Genome Institute, Washington University) containing pLKO.1‐puro plasmids were utilized for in vitro and in vivo murine MPNST cell experiments. Each construct was co‐transfected into HEK293T cells with the packaging plasmid pMDLg, pCMVg, and pREV using Mirus reagent (MilliporeSigma) to generate virus. After a 24‐hour infection, JW23.3 murine MPNST cells were subjected to selection media containing 2 µg/mL puromycin.

Three lentiviral sh*Tyk2* constructs were screened and used in vitro. The two constructs with the best knockdown were chosen for in vivo experiments.

### 2.3 CRISPR‐Cas9‐mediated *TYK2* knockout in MPNST 724 cells

We designed the sgRNA using the websitehttp://tools.genome-engineering.org.^19^

**Table:** 

gRNA #1‐1 CACCG GGCCCCACCTGGTAGGCATT	AAAC AATGCCTACCAGGTGGGGCC C
gRNA #1‐2 CACCG GCCATGGACAAGTGGGGGTT	AAAC AACCCCCACTTGTCCATGGC C
gRNA #2‐1 CACCG GCCCCACCTGGTAGGCATTC	AAAC GAATGCCTACCAGGTGGGGC C
gRNA #2‐2 CACCG GCCATGGACAAGTGGGGGTT	AAAC AACCCCCACTTGTCCATGGC C
gRNA #3‐1 CACCG GGAGACCTGGCTCATGAGGC	AAAC GCCTCATGAGCCAGGTCTCC C
gRNA #3‐2 CACCG GTGCATGGCGTCTGTGTGCG	AAAC CGCACACAGACGCCATGCAC C
gRNA #4‐1 CACCG ACTCAGCTTGATGAAGGGGC	AAAC GCCCCTTCATCAAGCTGAGT C
gRNA #4‐2 CACCG GGGCCTGGGCGCCCTCTCCA	AAAC TGGAGAGGGCGCCCAGGCCC C
gRNA #5‐1 CACCG CACCACCATCTTCCAAGCCA	AAAC TGGCTTGGAAGATGGTGGTG C
gRNA #5‐2 CACCG GGCCAGCGCCCTCAGCTACC	AAAC GGTAGCTGAGGGCGCTGGCC C
gRNA #6‐1 CACCG GCACACGCTGAACACTGAAG	AAAC CTTCAGTGTTCAGCGTGTGC C
gRNA #6‐2 CACCG GCAGCCCTGCCTGGGAGGAC	AAAC GTCCTCCCAGGCAGGGCTGC C

We constructed the pSpCas9n(BB)‐2A‐Puro (PX462)‐TYK2 plasmid by inserting the sgRNA into the plasmid. MPNST 724 cells (1 × 10^5^ cell/well) were transfected (Lipofectamine, Invitrogen, USA) with the paired plasmid with or without sgRNA. Cells were then puromycin selected for 72 hours. Two clones with the best knockout as demonstrated by western blot were chosen for analysis (#5 2 and #6 4 lines). One control clone was selected for further study as well (Control 8).

### 2.4 Western blot analysis

Cells were lysed in 1× Cell Lysis Buffer (Cell Signaling Technology, Danvers, MA). Protein content was quantified using the BCA kit (ThermoFisher Scientific, USA). Primary antibody incubations were performed at 4°C overnight (phospho‐STAT1(SC‐8394), phospho‐STAT3(SC‐8001‐R), STAT1(SC‐464), STAT3(SC‐23151), and Bcl2(SC‐7382) at 1:500, Santa Cruz Biotechnology, Dallas, Texas, USA; TYK2 (720124), GAPDH(AM4300), ThermoFisher Scientific, USA, Caspase3(9662) and Cleaved Caspase‐3(9661) at 1:1000; Cell Signaling Technology) diluted in 5% BSA TBS‐T. Secondary antibodies used included goat anti‐rabbit or goat anti‐mouse (Cell Signaling Technology) peroxidase‐conjugated antibodies (at 1:10000 dilution in 5% BSA TBS‐T). The protein levels were calculated by measuring the peak densitometric area (Image‐J). Each western blot analysis was repeated in triplicate. Phosphorylated and un‐phosphorylated proteins were run and detected on the same blot after stripping.

### 2.5 mRNA quantification

RNA was isolated using the TRIzol method (ThermoFisher Scientific, USA), then quantitated with the Nanodrop 2000 (ThermoFisher Scientific, USA). Reverse transcription reactions were performed with Superscript III (ThermoFisher Scientific, USA) according to the manufacturer's protocols. Quantitative PCR was performed in a CFX96 Touch™ Real‐Time PCR Detection System (Bio‐Rad, USA) using the SYBR® Green based qPCR kit (MilliporeSigma, St. Louis MO, USA). Primers used included:

**Table:** 

murine *Tyk2*	Forward 5′‐GTGACTCTAACCAGAGTCCCCATA‐3′,
	Reverse 5′‐CTGACCTTGGTACTTCTCCTGTG‐3′,
human *TYK2*	Forward 5′‐GACAGTCCATGAGAAGTACCAAGG‐3′,
	Reverse 5′‐CTCTAGACAGGAGTAAGGCACAC‐3′,
murine *Gapdh*	Forward 5′‐TCAACAGCAACTCCCACTCTTCCA‐3′,
	Reverse 5′‐ACCCTGTTGCTGTAGCCGTATTCA‐3′,
human *GAPDH*	Forward 5′‐TGTTGCCATCAATGACCCCTT‐3′,
	Reverse 5′‐CTCCACGACGTACTCAGCG‐3′.

These were synthesized by IDT (Integrated DNA Technologies, San Diego, CA). Relative gene expression was determined using CFX Manager^TM^ Software version 3.1 (Bio‐Rad, USA) by CFX96 Touch^TM^ and CFX96 Touch Deep well^TM^ Real‐Time PCR Detection Systems; ms‐*Gapdh* and hu‐*GAPDH* gene were used as reference genes for expression analysis.

### 2.6 Cell proliferation and cell death assays

Control sh*LacZ* JW23.3, sh*Tyk2* #1 JW23.3, sh*Tyk2* #2 JW23.3, and sh*Tyk2* #3 JW23.3, were cultured in standard conditions with 2 µg/mL puromycin, then plated at 5000 cells/well in 96‐well plates. For proliferation assays, cells were plated in phenol‐red free DMEM with 10% FBS. Cells were imaged every hour for 48 hours using the IncuCyte FLR imaging system (Essen Bioscience, Ann Arbor, MI, USA) and analyzed for quantitation using IncuCyte ZOOM Analysis Software (Essen Bioscience, Ann Arbor, MI) Phase images were used to determine percent confluence and subsequent wells were normalized to initial confluence. For cell death assays, 50nM TOTO^TM^‐3 iodide (ThermoFisher Scientific, USA) was added to the phenol‐red free media with reduced serum as a stress (5% FBS). Cells were imaged every hour for 48 hours using the IncuCyte FLR imaging system (Essen Bioscience, Ann Arbor, MI). For quantification of cell death, the TOTO^TM^‐3 iodide fluorescence was normalized to the confluency factor calculated from the phase of each respective well.

### 2.7 Tumor cell injections

For the subcutaneous model, a total of 2 × 10^6^ sh*LacZ* JW23.3, sh*Tyk2* #2 JW23.3, or sh*Tyk2* #3 JW23.3, tumor cells were injected into the dorsal surface of 5‐week‐old C57BL/6 ALBINO mice (Charles River Labs). Tumor volumes were measured weekly for the first 2 weeks and then every other day for the final week. For the left ventricular metastatic model, a total of 1 × 10^5^ sh*LacZ* JW23.3, sh*Tyk2* #2 JW23.3, or sh*Tyk2* #3 JW23.3, tumor cells were injected into the left ventricle of 5‐week‐old C57BL/6 ALBINO mice (Charles River Labs). These mice were followed by bioluminescence imaging (BLI) with weekly weights. (n = 5 mice per group). These experiments were all repeated independently and similar results were obtained. The Institutional Animal Care and Use Committee of Washington University has reviewed and approved our protocol for experiments utilizing animals. All animals are treated in compliance with IACUC policies.

### 2.8 Bioluminescence imaging (BLI)

BLI was performed on an IVIS 50 or IVIS Lumina (PerkinElmer; Living Image 4.3 or 3.2, 1 sec–5 minutes exposures, binning 2, 4, or 8; FOV 12 cm, f/stop1, open filter). In vivo bioluminescence was measured weekly post tumor cell injection following i.p. injection of D‐luciferin (150 mg/kg; Gold Biotechnology, Inc). Total photon flux (photons/second) was measured from fixed regions of interest over the entire mouse using Living Image 2.6.

### 2.9 Statistical analysis for in vivo and in vitro studies

In vivo tumor growth data GraphPad Prism Version 6 was used to perform a two‐way ANOVA analysis to determine statistical significance. Kaplan‐Meier analysis was used to determine statistical significance for overall survival. For all other in vivo and in vitro other experiments GraphPad Prism Version 6 was used to perform two‐tailed t‐tests to determine statistical significance.

### 2.10 Human tissue acquisition

Unstained slides were obtained from cases from individuals diagnosed with MPNSTs and treated at Washington University/St. Louis Children's Hospital NF Clinical Program (St. Louis, MO) under active Human Studies Protocols approved by the Institutional Review Boards in accordance with the 1964 Helsinki Declaration and its later amendments or comparable ethical standards. All human tumor samples were collected under an IRB approved protocol 201203042 and patients were appropriately consented.

### 2.11 Immunohistochemistry

Immunohistochemical staining was performed using a rabbit polyclonal antibody to TYK2 antibody at 1:1000 (ab39550; Abcam, Cambridge, Mass), Cleaved Caspase‐3 at 1:500 (Cell Signaling Technology) with citrate antigen retrieval on whole tissue sections from the most representative areas of the tumor, as well as from normal peripheral nerve. Breast carcinoma was used as a positive control for TYK2 staining. Images were acquired with the 40X objective using the Olympus DP70 Microscope Digital Camera and DP70‐BSW software. For TYK2 staining, tumors with strong immunostaining in >80% of the cells were scored as positive. Tumors with strong to moderate staining in 25%‐80% of cells were called moderate. Tumors with weak staining in <25% of cells or no immunostaining were deemed negative. No tumors called negative demonstrated any areas of moderate to strong staining.

### 2.12 Clinical data statistical analysis

Two‐way ANOVA analyses were performed to estimate the difference of tumor growth rates over time between *Tyk2* knockdown and control groups. The t‐tests were used to compare continuous variables between groups for In vitro experiments. The association between TYK2 protein expression and H3K27 me status was assessed by Fisher's Exact Test. Patient characteristics were compared between TYK2 normal and aberrant patients using χ2 test or Fisher exact test for categorical variables while non‐parametric Wilcoxon rank sum test was used for continuous variables. Kaplan‐Meier survival curves were generated for overall survival by TYK2 protein expression groups in MPNST. Patients were censored by date of death (Social Security Death Index, expiration note in chart or obituary) or date of last follow‐up. Overall survival is defined as date from diagnosis to date of death by any cause. The survival difference between groups was compared using the log‐rank test. Raw hazard ratios (HR) and 95% CI were estimated from univariate Cox model. All tests were two‐sided and *P*‐value ≤0.05 is considered statistically significant. Statistical analyses were performed with GraphPad Prism Version 6 and SAS (version 9.4; SAS Institute, Cary, NC).

## 3 RESULTS

### 3.1 TYK2 is expressed in the majority of MPNSTs

**Figure 1:**
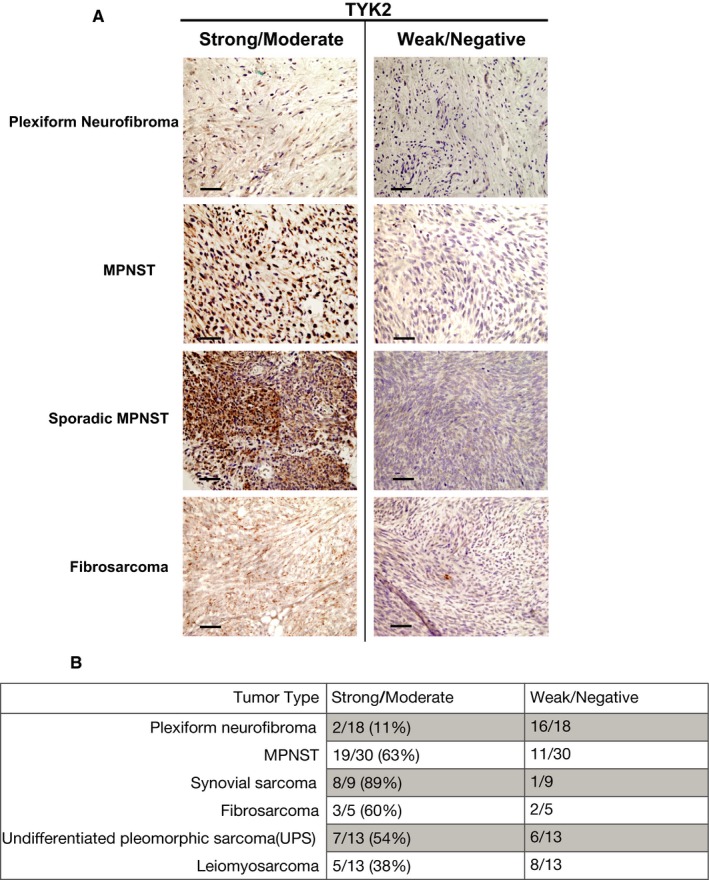
Tyrosine kinase 2 (TYK2) protein expression in malignant peripheral nerve sheath tumors (MPNSTs) and other sarcomas. TYK2 immunoreactivity was observed NF1‐associated and sporadic MPNSTs as well as other sarcomas. (A) Representative images of positive and negative staining for Plexiform Neurofibromas, NF1‐associated MPNSTs, sporadic MPNSTs, and Undifferentiated Pleomorphic Sarcoma (UPS), and fibrosarcoma. (B) Quantification of number of positive and negative cases observed for each type of tumor

We first set out to examine TYK2 expression in a set of MPNSTs and other sarcomas in order to determine if TYK2 expression could serve as a diagnostic biomarker for MPNSTs. We performed IHC on full tissue sections from 30 MPNSTs, 18 plexiform neurofibromas, 5 fibrosarcomas, 9 synovial sarcomas, 13 undifferentiated pleomorphic sarcomas, and 13 leiomyosarcomas. As can be seen in Figure 1, strong staining was observed in 63% of MPNSTs as well as 38%‐89% of other sarcomas, depending on the histologic subtype, but only 11% of benign plexiform neurofibromas. Table 1 depicts the clinical data associated with the MPNSTs in this study. A Fischer Exact Test was performed for each clinical parameter to see if there was any correlation between TYK2 expression. In this small sample size, no statistically significant correlation was observed. The polycomb repressive complex 2 (PRC2)/polycomb repressive complex 2 subunit (SUZ12) has recently been shown to play an important role in MPNST pathogenesis.^20^, ^21^ Given that PRC2 loss occurs in 60%‐70% of MPNSTs and affects transcriptional regulation, we sought to determine whether there was a correlation between PRC2 loss and TYK2 expression, which could suggest a link between these events. As a surrogate for PRC2/SUZ12 loss, we performed immunohistochemistry to examine H3K27 me3, a known downstream target of SUZ12. However, there was no correlation between these events in this cohort as determined by a Fischer Exact Test. Nonetheless, these data do suggest that TYK2 protein overexpression is prevalent in malignant tumors and could be targetable in MPNSTs as well as other sarcomas. Given that TYK2 is expressed at high levels in the majority of MPNSTs, we went on to look at the functional role in this subset of sarcomas.

**Table 1:** Clinical characteristics of MPNST patient

Characteristic	TYK2 positive (N = 18)	TYK2 Negative (N = 11)	Total (N = 29)	*P*‐value
Sex, n (%)				0.4497
Male	10 (56)	4 (36)	14 (48)	
Female	8 (44)	7 (64)	15 (52)	
Age at diagnosis, y				0.3419
Median	39	47	42	
Range	22‐60	18‐79	18‐79	
NF Status, n (%)				0.4486
NF1	8 (44)	3 (27)	11 (38)	
Sporadic	10 (56)	8 (73)	18 (62)	
Grade, n (%)				n/a
High	18 (100)	11 (100)	29 (100)	
Low	0 (0)	0 (0)	0 (0)	
Stage at diagnosis, n (%)				1.0
IV	3 (17)	1 (9)	4 (14)	
Other	15 (83)	10 (91)	25 (86)	
Site of tumor, n (%)				0.3312
Extremity	2 (11)	5 (45)	7 (28)	
Other	15 (83)	6 (55)	21 (72)	
H3K27 Trimethylation staining, n (%)				0.4328
Positive	11 (61)	4 (36)	15 (52)	
Negative	7 (39)	6 (54)	13 (45)	

### 3.2 Loss of TYK2 leads to increased cell death in vitro

**Figure 2:**
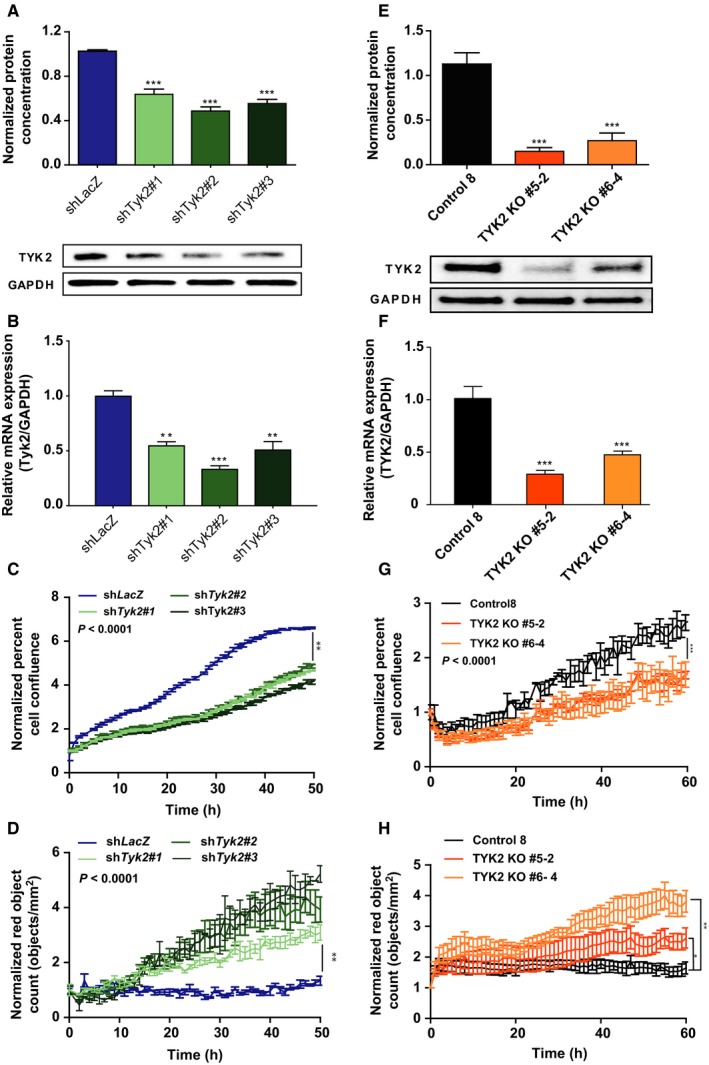
Loss of *Tyk2*/TYK2 in JW23.3 murine MPNST and MPNST 724 cells leads to increased cell death. sh*Tyk2*‐infected JW23.3 cell lines and control sh*LacZ*: (A) Western blot densitometry analysis measuring TYK2 protein. (B) Relative Expression of *Tyk2* mRNA compared to *Gapdh*. (C) An Incucyte cell proliferation assay measuring confluence over time. (D) An Incucyte death assay measuring TOTO^TM^‐3 iodide fluorescence as an indicator of death over time. *TYK2* knockout MPNST 724 cells and scramble control: (E) Western blot densitometry analysis of TYK2 protein. (F) Relative Expression of *TYK2* mRNA compared to GAPDH. (G) An Incucyte cell proliferation assay measuring confluence over time. (H) An Incucyte death assay measuring TOTO^TM^‐3 iodide fluorescence as an indicator of death over time. (*P < 0.05, **P < 0.01, ***P < 0.001)

**Figure 3:**
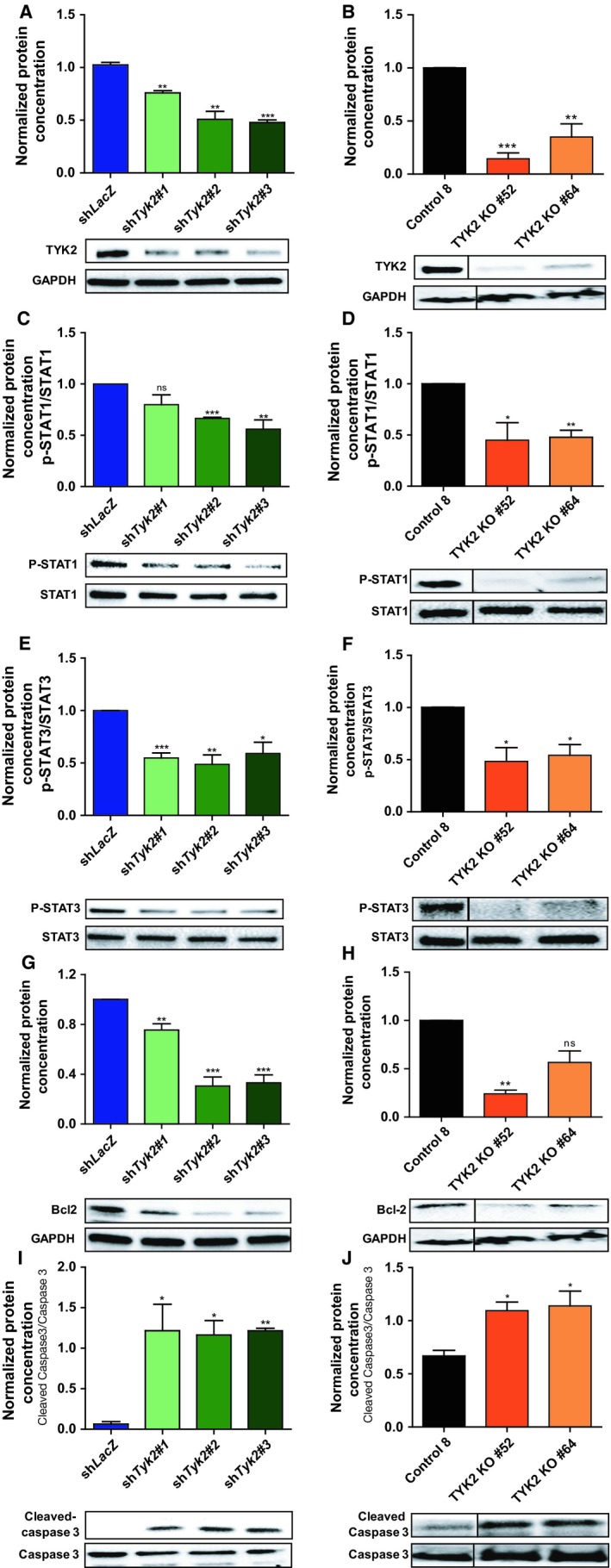
Knockdown of *Tyk2*/*TYK2* in JW23.3 murine MPNST and MPNST 724 cells affects expression of downstream targets. Western blot densitometry analysis on downstream targets of *Tyk2*/TYK2. Murine JW23.3 cell lines are depicted in blue/green, human MPNST 724 cell line are depicted in black/orange. (A and B) levels of TYK2 normalized to GAPDH (C and D) levels of p‐STAT1 normalized to total STAT1. (E and F) levels of p‐STAT3 normalized to total STAT3. (G and H) levels of Bcl‐2 normalized to GAPDH (I and J) levels of Cleaved Caspase‐3 normalized to Caspase 3 levels. (*P < 0.05, **P < 0.01, ***P < 0.001) All experiments were done in triplicate. Representative blots are shown

To explore the function of Tyk2 in vitro, we utilized JW23.3 *Nf1/Tp53*‐mutant NPcis murine MPNST cells engineered to express firefly luciferase. Following *Tyk2* shRNA‐mediated knockdown, there was reduced expression of Tyk2 by western blot compared to control sh*LacZ* virus infection (Figure 2A), as well as decreased expression at an mRNA level (Figure 2B). Reduced Tyk2 expression was associated with decreased cell confluence over time (Figure 2C, Supplemental Figure S1A) as well as increased cell death as assessed by increased incorporation of TOTO^TM^‐3 iodide, a fluorescent dye binding to cell‐free DNA (Figure 2D, Supplemental Figure S1B) compared to control cells. To ensure that these data were not an artifact of a single cell line, we next used the CRSIPR/Cas9 system to knockout *TYK2* in human MPNST 724 cells and demonstrated similar findings (Figure 2E‐H, Supplemental Figure S1C and D). To explore potential downstream targets by which TYK2 could be exerting its effects on cell death in MPNSTs, we first examined phosphorylation status of several downstream STAT proteins, as these are known targets for Tyk2.^16^, ^17^ As can be seen in Figure 3, upon knockdown of *Tyk2* (Figure 3A and B) there was a significant reduction in levels of phospho‐STAT1 and phospho‐STAT3 in both murine and human cell lines (Figure 3C‐F). Additionally, there was a decrease in levels of the anti‐apoptotic protein, Bcl‐2 (Figure 3G and H), another protein shown to be regulated by TYK2 in other models.^16^ This was associated with increased levels of Cleaved Caspase‐3 (Figure 3I and J).

### 3.3 Loss of TYK2 leads to decreased tumor burden in vivo

**Figure 4:**
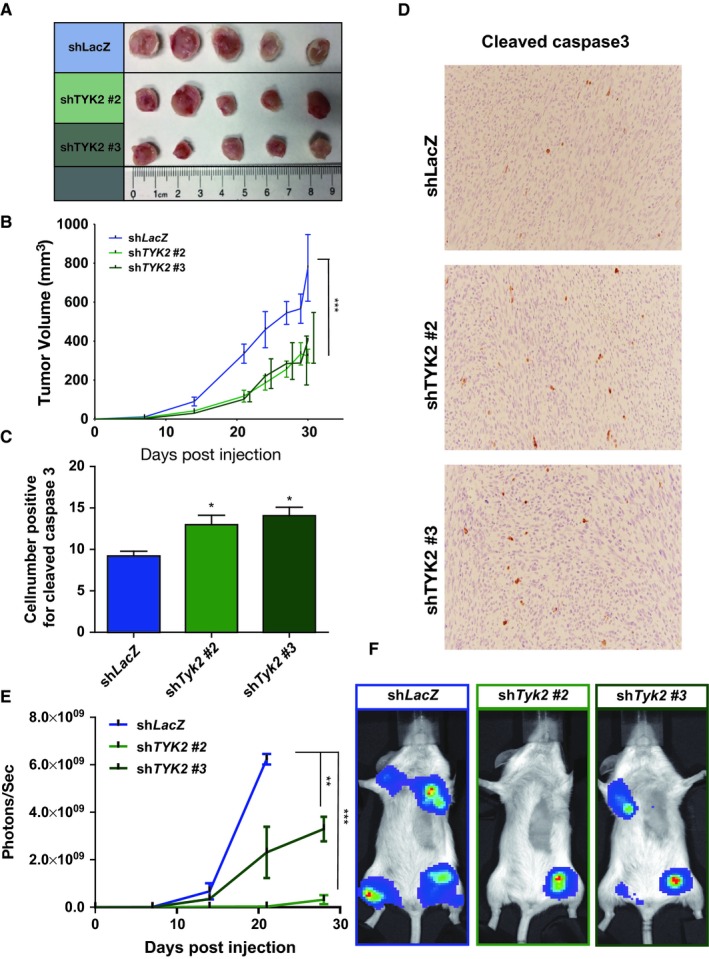
shRNA‐mediated knockdown of *Tyk2* in JW23.3 murine MPNST cells leads to decreased tumor growth in vivo. (A) Dissected tumors from subcutaneous injection of sh*Tyk2*‐infected JW23.3 cell lines and control sh*LacZ*. (B) Quantification of subcutaneous tumor volume over time. (C) Quantification of the number of Cleaved Caspase‐3 positive cells from immunohistochemistry of harvested subcutaneous tumors of sh*Tyk2*‐infected JW23.3 cell lines and control sh*LacZ*. (D) Representative images of Cleaved Caspase‐3 immunohistochemistry. (E) Tumor burden over time as measured by photon flux in mice injected intraventricularly. (F) Representative images of mice following intraventricular injection. (n = 5 for all groups; *P < 0.05, **P < 0.01, ***P < 0.001)

We next chose *shTyk2* #2 and *shTyk2* #3 to use to determine the effects of *Tyk2* knockdown in vivo using the JW23.3 *Nf1/Tp53*‐mutant NPcis murine MPNST cells. These lines were chosen as they produced the greatest and most consistent knockdown of *Tyk2*. In a subcutaneous model of tumor growth, we observed decreased tumor volume over time in the setting of *Tyk2* knockdown (Figure 4A and B). Additionally, tumor sections from these mice demonstrated increased numbers of Cleaved Caspase‐3 positive cells (Figure 4C and 4D), demonstrating that at least a subset of cells are going through apoptosis. Furthermore, in a left ventricular tumor injection model of tumor dissemination and metastasis, we observed decreased tumor burden over time in mice injected with *Tyk2*‐deficent cells compared to mice injected with control cells (Figure 4E and 4F). This was associated with decreased weight loss (Supplemental Figure S2A) and increased overall survival (Supplemental Figure S2B).

## 4 DISCUSSION

MPNSTs are aggressive sarcomas with limited treatment options and a dismal overall survival. As such, better therapies are desperately needed for these tumors. Prior work from our laboratory identified potential activating mutations in *TYK2* in one‐third of MPNSTs examined. TYK2, a member of the Janus Kinase family of proteins has been shown to play a role in immune surveillance and host response in the setting of infection, autoimmune disorders, and malignancy.^22^, ^23^, ^24^, ^25^ More recent cancer genomic studies have identified activating mutations in *TYK2* within cancer cells, implicating a cell intrinsic role for TYK2 in promoting cancer progression.^9^, ^25^, ^26^, ^27^ Based on these genomic studies, we have begun to explore the role of TYK2 in MPNST pathogenesis. The current study raises several important points. First, we have demonstrated the TYK2 is present in the majority of MPNSTs. In this cohort, we performed an analysis to see if there was any correlation between TYK2 expression and overall survival or any other clinical parameter. Unfortunately, we did not see any statistically significant associations in this set. Future work is aimed obtaining a larger sample set through collaboration with several other NF centers. Nonetheless, the strong staining in the majority of MPNSTs suggests that TYK2 could be a therapeutic target for a large proportion of these tumors. While, we had initially hoped that expression of TYK2 could serve as a potential biomarker distinguishing MPNSTs from other soft tissue sarcomas, we observed high expression in 38%‐89% of other sarcomas as well. As such, TYK2 expression is unlikely to serve as a diagnostic biomarker for MPNSTs, but these data do suggest that TYK2 could be a therapeutic target in other sarcoma subtypes as well. Future work is aimed at evaluating this possibility.

Second, we have demonstrated that decreased expression of TYK2 in both human and mouse MPNST cells leads to decreased cell survival, decreased activation of STAT1 and 3, and decreased levels of Bcl2 in vitro. Interestingly, STAT3 has been previously implicated in MPNST pathogenesis.^28^ Taken together, these data suggest that the TYK2 pathway could serve as a therapeutic target in MPNSTs. Future work is aimed at dissecting the upstream components in this pathway and identifying other downstream targets that could be involved in controlling TYK2‐dependent cell survival. Third, we demonstrate that reduced levels of Tyk2 leads to decreased tumor growth in subcutaneous and left ventricular models of tumor inoculation in a murine MPNST. Interestingly, while there were far more Cleaved Caspase‐3 positive cells in the *Tyk2* knockdown tumors compared to control, there were still a large number of cells that were not Cleaved Caspase‐3 positive. Despite this fact, there was a very significant decrease in tumor burden, suggesting that there may be other cell death mechanisms at play in addition to apoptotic cell death. Nonetheless, the mice injected with Tyk2‐deficient cells exhibited less weight loss and had an increased overall survival, further supporting targeting this pathway in MPNSTs. Future studies will be aimed at better understanding the mechanisms at play in vivo. As we move forward with evaluating this pathway as a viable therapeutic target, another important aspect of TYK2 signaling that cannot be ignored is that TYK2 does not act alone, but is rather in a receptor complex in which either JAK1 or JAK2 is also involved. Thus, drug development strategies will benefit from understanding which of the other JAK family members are important in MPNST biology. Future work will be aimed at identifying the most effective inhibitor from in vitro studies and then evaluating the in vivo efficacy of the most promising TYK2 inhibitors to test as a single agent and in combination therapies.

## CONFLICT OF INTEREST

The authors declare no potential conflicts of interest.

## AUTHOR CONTRIBUTIONS

Performed experiments: WQ, AG, XZ, CZ. Analyzed data: WQ, AG. Supplied Samples: JS, ACH. Writing of manuscript: WQ, ACH. Guidance and editing: XB, ACH.
