# Supplementary material for: TYK2 promotes malignant peripheral nerve sheath tumor progression through inhibition of cell death
Source: Cancer Med. 2019 Jul 6;8(11):5232–41. doi: 10.1002/cam4.2386 (PMC6718590; doi:10.1002/cam4.2386)
Supplement: Supplementary file 1 [file CAM4-8-5232-s001.pdf]

Supplemental Material for: TYK2 Promotes Malignant Peripheral Nerve Sheath Tumor Progression through Inhibition of Cell Death

Wenjing Qin 1, 2, Abigail Godec1, Xiaochun Zhang1, Cuige Zhu1, Jieya Shao 1, 3 Yu Tao 1, Xianzhang Bu 2, Angela C Hirbe 1, 3

1Division of Oncology, Department of Internal Medicine, Washington University School of Medicine, St. Louis, MO; 2 School of Pharmaceutical Sciences, Sun Yat-Sen University, Guangzhou 510006, China; 3Siteman Cancer Center, Washington University School of Medicine, Saint Louis, MO

Corresponding Author:
Angela C Hirbe: Division of Medical Oncology, Box 8076 660 S. Euclid Avenue, Washington University School of Medicine, St. Louis, MO 63110. hirbea@wustl.edu Phone: 314-747-3096; Fax: 314-362-7086

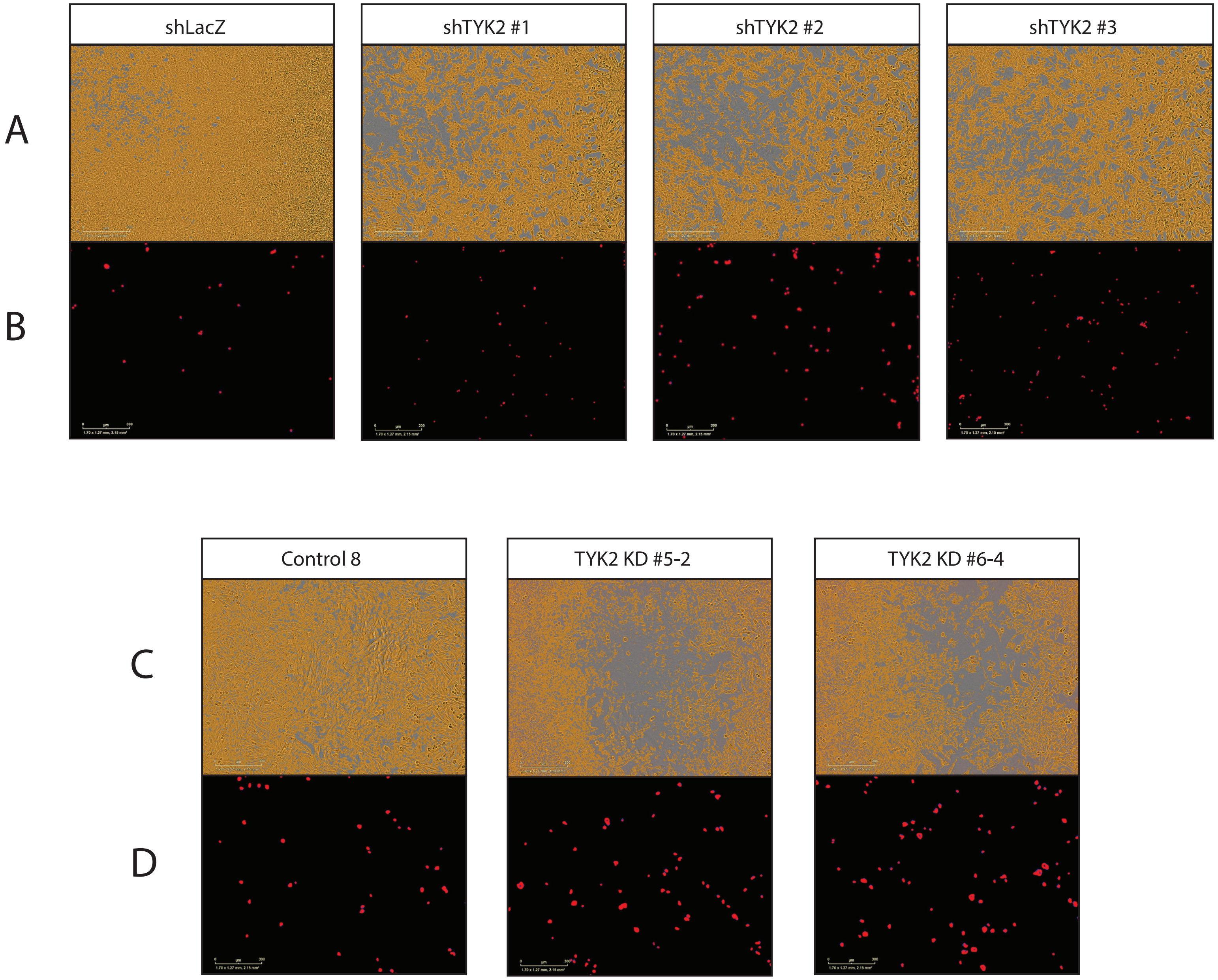

Supplemental Figure 1. Reduction of TYK2 expression in cell proliferation and death.
All images are from Incucyte assays taken at 48 hours. (A) Representative phase images from cell proliferation assay for JW23.3 cells, confluency is depicted in orange. (B) Representative images from cell death assay for JW23.3 cells, fluorescence of TOTO-3 iodide depicted in red. (C) Representative phase images from cell proliferation assay for MPNST 724 cells, confluency is depicted in orange. (B) Representative images from cell death assay for MPNST 724 cells, fluorescence of TOTO-3 iodide depicted in red.

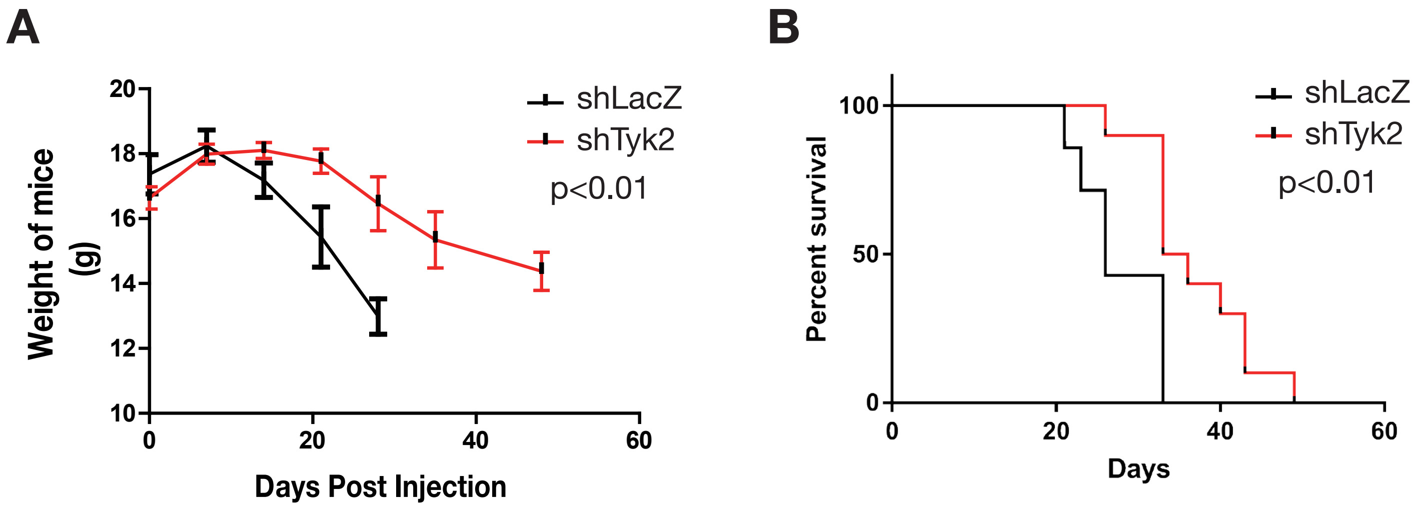

Supplemental Figure 2. shRNA-mediated knockdown of Tyk2 in JW23.3 a murine metastasis model led to decreased weight loss and increased overall survival.
(A) Weight of mice over time in intraventricular injection metastasis model. (B) Kaplan Meier curve from intraventricular injection metastasis model. Mice injected with Tyk2 deficient cells, were grouped for survival analysis (n=12, control n= 6; $p<0.01$).
